# Benzyl isothiocyanate suppresses development and metastasis of murine mammary carcinoma by regulating the Wnt/β-catenin pathway

**DOI:** 10.3892/mmr.2022.12823

**Published:** 2022-08-10

**Authors:** Bei Xie, Lei Zhao, Lanlan Guo, Hang Liu, Siyu Fu, Wenjuan Fan, Li Lin, Jing Chen, Bei Wang, Linlan Fan, Hulai Wei

Mol Med Rep 20: 1808–1818, 2019; DOI: 10.3892/mmr.2019.10390

Subsequently to the publication of the above paper, an interested reader drew to the authors’ attention that the ‘1 µM/Invasion’ and the ‘2.5 µM/Migration’ panels shown in Fig. 3B on p. 1814 appeared to contain overlapping sections of data, such that they were potentially derived from the same original source, where these panels was intended to show the results from differently performed experiments. The authors have re-examined their original data, and realize that Fig. 3B was inadvertently assembled incorrectly; specifically, the ‘2.5 µM/Migration’ panel was selected from the wrong data group.

The revised version of Fig. 4, now containing the correct data for the ‘2.5 µM/Migration’ experiment in Fig. 3B, is shown on the next page. Note that this error did not adversely affect either the results or the overall conclusions reported in this study. All the authors agree with the publication of this corrigendum, and are grateful to the Editor of *Molecular Medicine Reports* for allowing them the opportunity to publish this. They also wish to apologize to the readership of the Journal for any inconvenience caused.

## Figures and Tables

**Figure 3.**
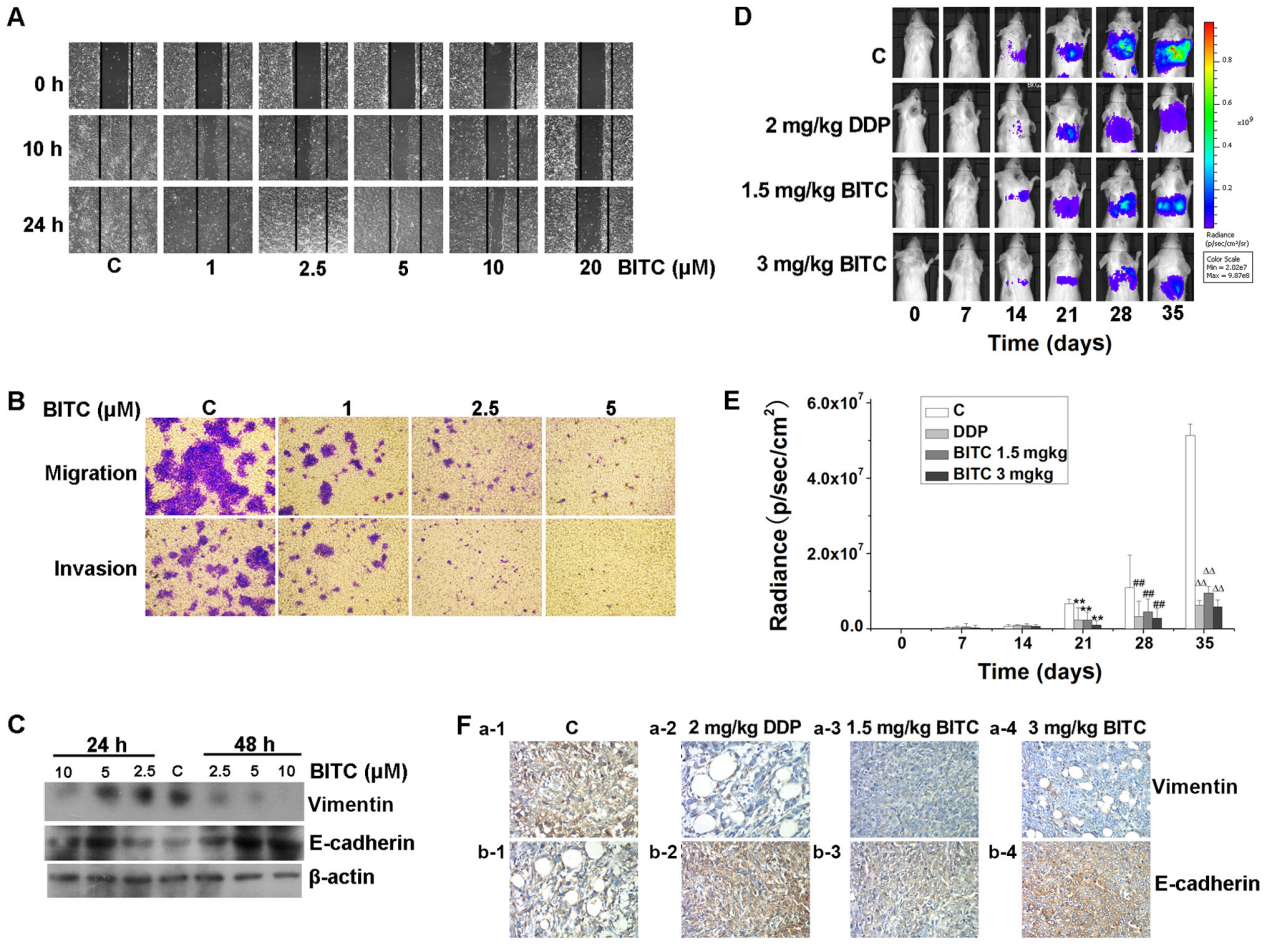
BITC inhibits the migration, invasion and metastasis of 4T1-Luc cells. (A) BITC inhibited the migration of 4T1-Luc cells. 4T1-Luc cells were treated with BITC as indicated and subjected to a wound healing assay (×100). (B) BITC inhibited the migration and invasion of murine mammary carcinoma cells. 4T1-Luc cells were cultured in Transwell chambers coated with or without Matrigel for 24 h, in the presence of the indicated concentrations of BITC (×40). (C) BITC inhibited cellular migration by regulating the expression levels of vimentin and E-cadherin. 4T1-Luc cells were treated with various concentrations of BITC as indicated for 24 and 48 h. Total protein lysates were immunoblotted for E-cadherin and vimentin expression. β-actin was used as the internal control. (D) Inhibitory effects of BITC on the metastasis of murine mammary carcinoma in mice was monitored using an optical *in vivo* bioluminescence imaging system every 7 days (n=8 mice/group). (E) Quantification of radiance recorded from the tumors were analyzed. **P<0.01 vs. C (21 days); ##P<0.01 vs. C (28 days); ∆∆P<0.01 vs. C (35 days). (F) Tumor sections were assessed via immunohistochemistry to determine the expression of E-cadherin and vimentin. The expression of E-cadherin was upregulated, whereas that of vimentin was downregulated in BITC-treated mice compared with those in the control group (magnification, ×400). BITC, benzyl isothiocyanate; C, control; DDP, cisplatin.

